# Prognostic value of computed tomography-derived myocardial extracellular volume in aortic stenosis: a meta-analysis of all-cause mortality and heart failure hospitalization

**DOI:** 10.1093/ehjopen/oeaf007

**Published:** 2025-01-25

**Authors:** Jin Kirigaya, Shingo Kato, Kensuke Matsushita, Nobuyuki Horita, Daisuke Utsunomiya, Kiyoshi Hibi

**Affiliations:** Division of Cardiology, Yokohama City University Medical Center, 4-57, Urafune-cho, Minami-ku, Yokohama, Kanagawa 232-0024, Japan; Department of Diagnostic Radiology, Yokohama City University Graduate School of Medicine, 3-9 Fukuura, Kanazawa-ku, Yokohama 236-0004, Japan; Division of Cardiology, Yokohama City University Medical Center, 4-57, Urafune-cho, Minami-ku, Yokohama, Kanagawa 232-0024, Japan; Chemotherapy Center, Yokohama City University Hospital, 3-9 Fukuura, Kanazawa-ku, Yokohama 236-0004, Japan; Department of Diagnostic Radiology, Yokohama City University Graduate School of Medicine, 3-9 Fukuura, Kanazawa-ku, Yokohama 236-0004, Japan; Division of Cardiology, Yokohama City University Medical Center, 4-57, Urafune-cho, Minami-ku, Yokohama, Kanagawa 232-0024, Japan; Department of Cardiology, Yokohama City University School of Medicine, 3-9 Fukuura, Kanazawa-ku, Yokohama 236-0024, Japan

**Keywords:** Aortic stenosis, Computed tomography, Extracellular volume fraction, Prognostic value

## Abstract

**Aims:**

Pre-existing myocardial fibrosis before aortic valve replacement (AVR) is a major cause of postoperative heart failure (HF). Evaluation of fibrosis by computed tomography extracellular volume (CT-ECV) may allow risk stratification for patients with severe aortic stenosis (AS) scheduled for transaortic AVR (TAVR) or surgical AVR (SAVR). We performed a meta-analysis to determine the prognostic value of CT-ECV for the prediction of adverse events in patients with severe AS scheduled for AVR.

**Methods and results:**

Electronic database searches of PubMed, Web of Science Core Collection, Cochrane advanced search, and EMBASE were performed. A comprehensive literature review was conducted to examine the association between CT-ECV and prognosis in patients with severe AS who underwent AVR. The diagnostic performance of CT-ECV for predicting composite adverse events (all-cause death and hospitalization for HF) was assessed using a pooled odds ratio (OR). Data from 902 patients with severe AS were extracted from six studies, including 881 TAVR and 21 SAVR cases. The pooled OR of abnormal CT-ECV for predicting adverse events was 4.53 [95% confidence interval (CI): 3.13–6.57 (*I*^2^ = 10%, *P* for heterogeneity = 0.50)]. We performed an OR meta-analysis on five studies with only TAVR cases (*n* = 807). The pooled OR of abnormal CT-ECV for predicting adverse events in TAVR patients was 4.85 [95% CI: 3.26–7.21 (*I*² = 0%, *P* < 0.001)].

**Conclusion:**

Considering the high prognostic ability and versatility of CT-ECV, it may be used to predict postoperative adverse events in patients with severe AS who underwent AVR.

## Graphical abstract

**graphical abstract:**
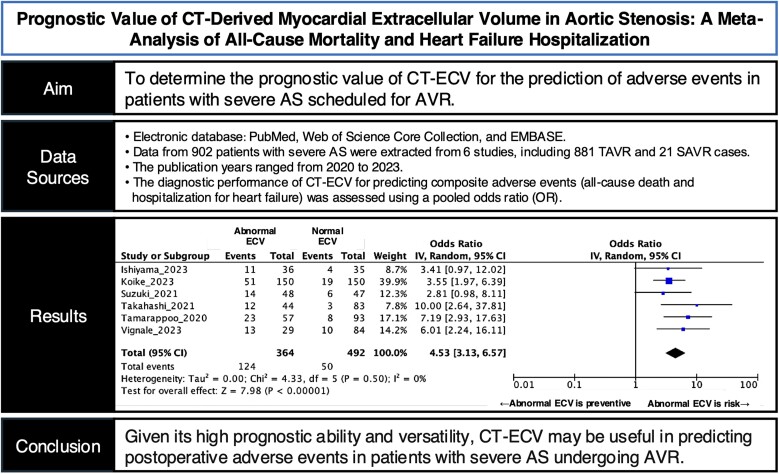

## Introduction

Transcatheter aortic valve replacement (TAVR) emerges as a novel therapeutic approach for patients with severe aortic stenosis (AS). Transcatheter aortic valve replacement indications have expanded significantly in the last 15 years,^1–3^ moving from only high-risk and inoperable patients to intermediate- and low-risk patients.^4,5^ Post-TAVR and post-surgical AVR (SAVR) patients have a residual risk of developing heart failure (HF) and cardiovascular death, even after successful valve replacement.^6,7^ While multiple factors contribute to the risk of residual HF, pre-existing myocardial fibrosis is presumed to be one of the important causes.^8^ Left ventricular (LV) fibrosis has also been linked to cardiovascular death, including arrhythmia-related mortality.^9,10^ Thus, assessing myocardial fibrosis is paramount in determining the appropriate treatment approach after AVR and predicting patient outcomes.

Lately, computed tomography (CT) imaging has been utilized to evaluate extracellular volume (ECV), demonstrating reliability on par with cardiac magnetic resonance (CMR)-derived ECV.^11^ Computed tomography ECV is particularly useful in detecting AS-related fibrosis because it only requires additional late iodine enhancement CT imaging, which is quick and can easily be incorporated into CT studies before the AVR procedure. Evaluation of pre-existing myocardial fibrosis in AS by CT-ECV may identify a high-risk population that may develop HF after AVR. Nonetheless, CT-ECV has not yet achieved widespread usage, and its effectiveness remains insufficiently studied.

Consequently, we have conducted a meta-analysis of studies concerning the prognostic value of CT-ECV in patients with AS scheduled for AVR.

## Methods

### Search strategy and selection criteria

We utilized the methods recommended by the Cochrane Collaboration and adhered to the reporting criteria outlined in the 2020 Preferred Reporting Items for Systematic Review and Meta-Analysis guideline.^12^ A database search was conducted using PubMed, Web of Science Core Collection, Cochrane advanced search, and EMBASE electronic database on 24 April 2024. The following keywords were used: computed tomography, CT, extracellular volume, ECV, CT-ECV, aortic stenosis, and AS (see Supplementary material online, *Table S1*). Eligibility criteria are all papers presenting CT-ECV values for AS that are searchable by keyword. Data extraction included both prospective and retrospective studies that contained CT-ECV data for AS. We excluded case reports, animal studies, and papers not in English as part of our exclusion criteria. We also excluded studies that included cases who underwent balloon aortic valvuloplasty without subsequent AVR, as balloon aortic valvuloplasty alone for severe AS patients is typically associated with poor prognosis.^13,14^ After screening all titles and abstracts from the search results, potentially relevant studies underwent full review for eligibility by two reviewers (J.K. and S.K.). A third reviewer resolved any discrepancies. Our study protocol was registered with the University Medical Information Network (registration number: UMIN000054148). Institutional review board approval was not required for this meta-analysis since it did not involve clinical patient information.

### Outcome measures

The primary outcome of this meta-analysis was to determine the prognostic impact of CT-ECV in patients with severe AS who underwent AVR. To calculate the pooled odds ratio (OR), the number of normal and abnormal CT-ECV events was investigated. Adverse events were defined as hard endpoints: all-cause death and hospitalization for HF. Two reviewers extracted study characteristics, such as author name, publication year, country of origin, definition, patient disease, age, sex, and CT-ECV values.

### Assessment of the risk of bias

We used the Newcastle-Ottawa Quality Assessment Scale and case-control studies to evaluate the risk of bias.^15^ To assess publication bias, funnel plots were presented for OR for the composite endpoint in AS patients.

### Statistical analysis

A random-effects model meta-analysis was performed using RevMan 5.41 (Cochrane Collaboration, London, UK). The OR meta-analysis of AS was conducted using the general inverse variance method. To investigate the prognostic value of CT-ECV in the patients who underwent TAVR, we performed the OR meta-analysis in the five studies that included only TAVR patients.^16–21^ Only Suzuki *et al*.’s study included patients who underwent SAVR, and we could not assess the OR for the outcomes of TAVR and SAVR separately. To assess potential sources of heterogeneity, we performed subgroup analyses. First, we conducted an OR meta-analysis to compare the prognostic value of CT-ECV between single-energy CT^19,21^ and dual-source CT.^16–19^ Given that three of the six included studies were conducted in Japan, we also analysed studies conducted in Japan^18,20,21^ separately from those conducted in non-Japanese^16,17,19^ facilities to examine regional differences. Finally, considering the variability in follow-up durations among the studies, we divided them into two groups based on follow-up duration: those with longer follow-up periods (e.g. Suzuki *et al*. and Koike *et al*.)^18,19^ and those with shorter follow-up periods.^16,17,20,21^ Heterogeneity was indicated by *I*², with 0% showing no heterogeneity and 100% indicating substantial heterogeneity. The funnel plot was statistically evaluated using Begg's test. A *P*-value of <0.10 was considered statistically significant for Begg's test, while a *P*-value of <0.05 was considered statistically significant for other statistical analyses.

## Results

**Figure 1:**
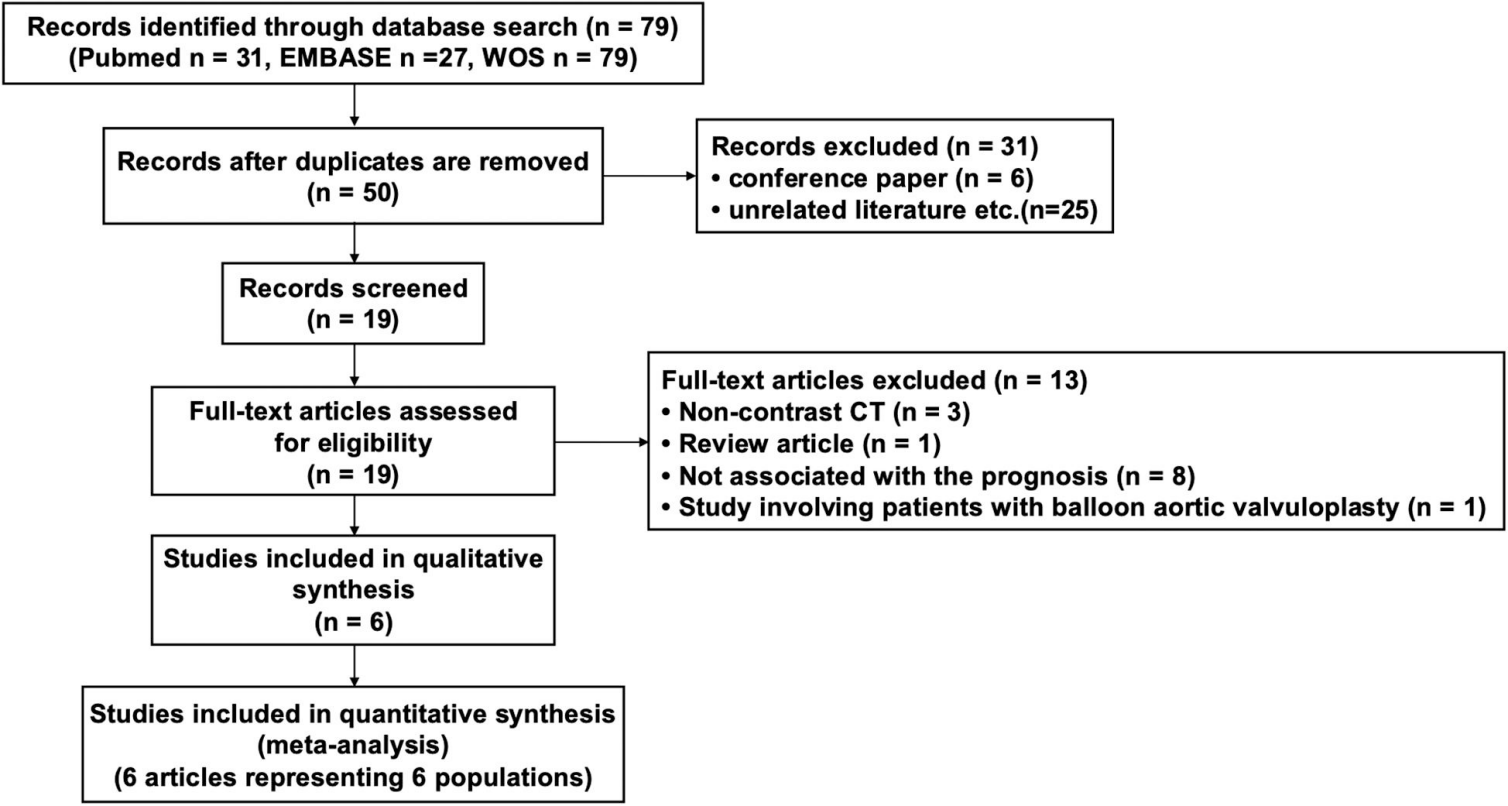
Preferred reporting items for systematic reviews and meta-analyses flow diagram. CT, computed tomography; EMBASE, Excerpta Medica Database; WOS, Web of Science.

Finally, six eligible publications^16–21^ were selected from 19 candidate papers (*Figure 1*). *Table 1* summarizes the characteristics of the included studies. These six publications presented data on 902 patients. The number of cases for each AVR was 881 for TAVR and 21 for SAVR. The publication years ranged from 2020 to 2023. About the technology of the CT machine, two investigations used a single-energy CT,^20,21^ whereas four used a dual-source CT.^16–19^ In the study by Suzuki *et al*.,^18^ the cohort comprised candidates for AVR, with 74 undergoing TAVR and 21 receiving SAVR. Five of the remaining six studies focused solely on patients who underwent TAVR.^16,17,19–21^ One of these studies was exclusively dedicated to patients with low-flow, low-gradient AS.^17^

**Table 1:** Characteristics of included studies

Author year	Country	Study design	*n*	Age, years	Male, %	Inclusion criteria	AVR procedure	CT scanner	Time from contrast injection to imaging	Analysis method
Tamarappoo_2020	United States of America	Retrospective single-centre study	150	81 ± 10	60	LF-LG AS with TAVR	TAVR 150	DSCT	5	Mean of the septum and lateral wall
Suzuki_2021	Japan	Retrospective single-centre study	95	84.0 ± 5.0	75	Severe AS candidate for AVR	SAVR 21 TAVR 74	DSCT	5	16 segment model
Ishiyama_2023	Japan	Prospective single centre	71	84.1 ± 5.2	40.8	Severe AS underwent TAVR	TAVR 71	320 detector row CT, single energy	5	16 segment model
Takahashi_2021	Japan	Retrospective single-centre study	127	84 ± 5	39	Severe AS underwent TAVR	TAVR 127	256 detector row CT, single energy	6	16 segment model
Vignale_2023	Italy	Prospective single centre	159	82 (79–85)	48	Severe AS underwent TAVR	TAVR 159	DSCT	5	Interventricular septum
Koike_2023	United States of America	Retrospective single-centre study	300	80.0 ± 9.4	55	Severe AS underwent TAVR	TAVR 300	DSCT	3	Interventricular septum

BMI, body mass index; CABG, coronary artery bypass grafting; CAD, coronary artery disease; DSCT, dual-source computed tomography; DLP, dyslipidaemia; DM, diabetes mellitus; HR, heart rate; HT, hypertension; LF-LG, low flow low gradient.

The quality ratings of the studies for the assessment of risk of bias are summarized in Supplementary material online, *Table S2*. Overall, five of six studies (83%) were rated as high quality (scoring >80% on the quality scales), one of six studies (17%) was rated as moderate quality (scoring between 50% and 80% on the quality scales), and zero of six studies (0%) were rated as low quality (<50% score on the scales). The evaluation of the funnel plot of the OR for the composite endpoint in patients with AS was performed. The *P*-value for Begg’s test was 0.464, indicating that significant publication bias was undetected (see Supplementary material online, *Figure S1*).

### Association between computed tomography extracellular volume and composite adverse events

**Figure 2:**
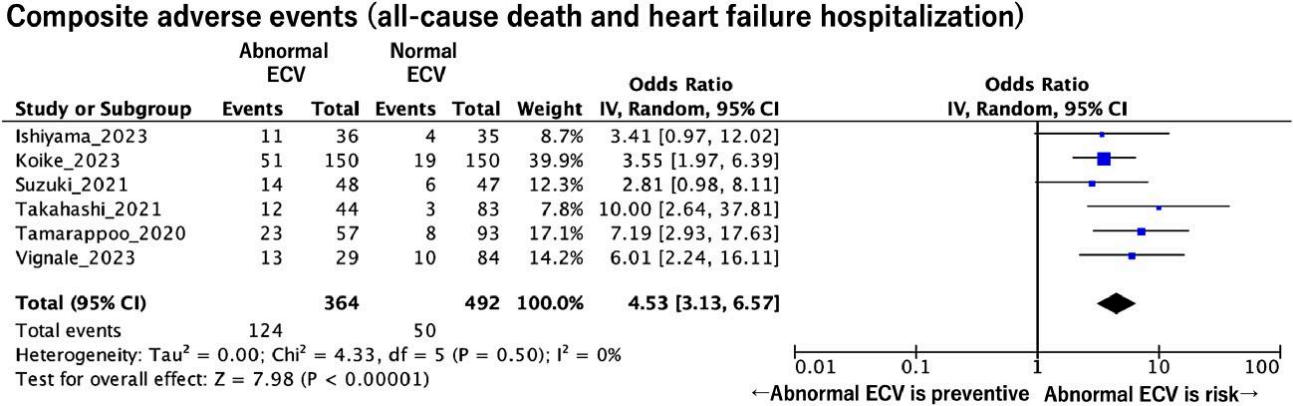
The pooled odds ratio of abnormal computed tomography extracellular volume for predicting composite adverse events (all-cause death and heart failure hospitalization). CI, confidence interval; ECV, extracellular volume.

All studies defined the outcome as a composite adverse event. Supplementary material online, *Table S3* summarizes definitions for adverse events, a follow-up period, and a cut-off value of CT-ECV for predicting adverse events. The cut-off values for CT-ECV were determined using the median in three studies,^18–20^ Youden index in two studies,^16,21^ and Liu index in one study.^17^ A composite adverse event was defined as the total number of all-cause deaths and hospitalizations for HF.^16–21^ Extracellular volume cut-off value in predicting adverse events ranged from 28% to 33%. A meta-analysis revealed that a higher CT-ECV was significantly associated with a higher composite adverse events rate [OR = 4.53 per unit increase; 95% confidence interval (CI): 3.13–6.57; *P* < 0.001; *I*^2^ = 10%; *P* for heterogeneity = 0.50] (*Figure 2*).

**Figure 3:**
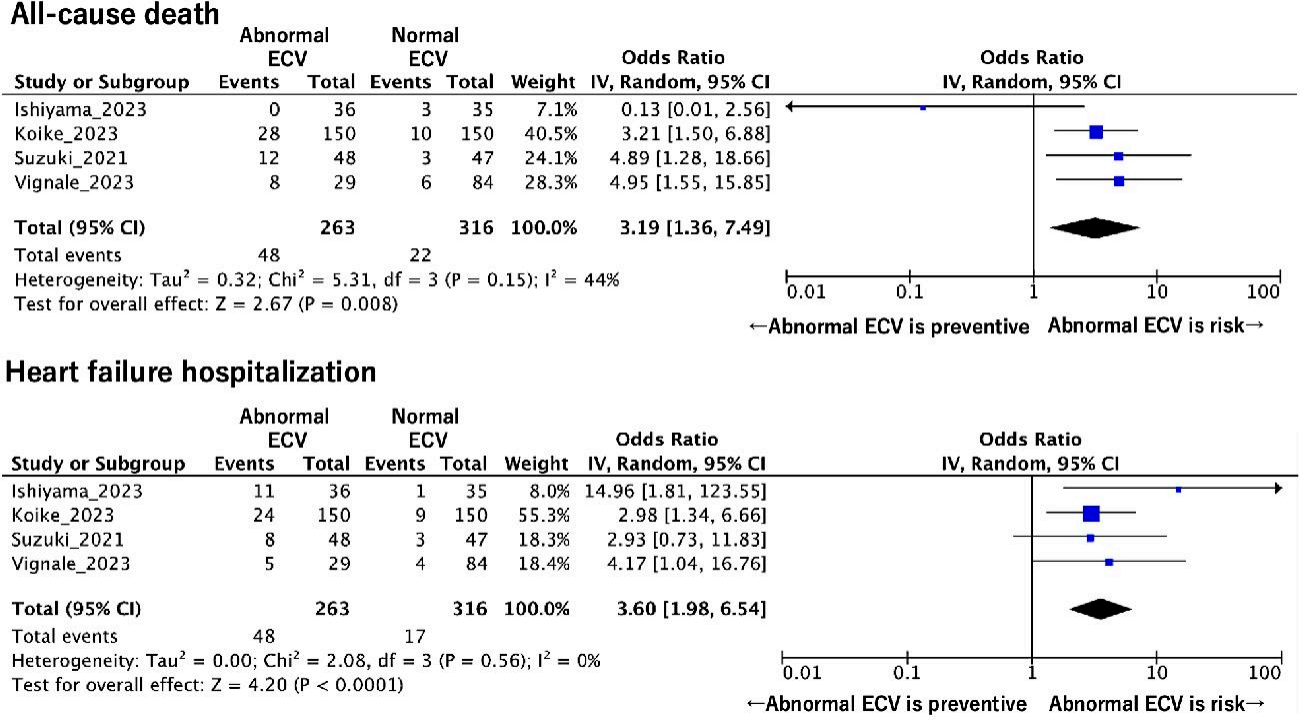
The pooled odds ratio of abnormal computed tomography extracellular volume for predicting all-cause death and heart failure hospitalization. CI, confidence interval; ECV, extracellular volume.

Four studies indicated the ORs of all-cause mortality and HF hospitalization separately.^16,18–20^ These data were used to create the pooled ORs of abnormal CT-ECV values to predict all-cause mortality and HF hospitalization separately. The pooled OR of abnormal CT-ECV for predicting all-cause mortality was 3.19 per unit increase (95% CI: 1.36–7.49; *P* = 0.008; *I*^2^ = 45%; *P* for heterogeneity = 0.15) (*Figure 3*). The pooled OR of abnormal CT-ECV for predicting hospitalization for HF was 3.60 per unit increase (95% CI: 1.98–6.54; *P* < 0.001; *I*^2^ = 0%; *P* for heterogeneity = 0.56).

### Prognostic value of computed tomography extracellular volume in patients who underwent transcatheter aortic valve replacement

**Figure 4:**
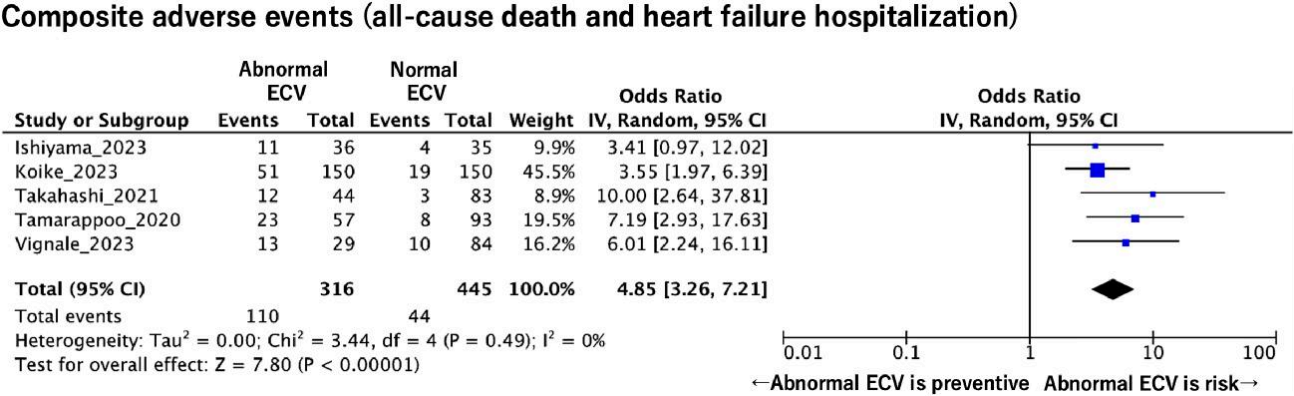
The pooled odds ratio of abnormal computed tomography extracellular volume for predicting composite adverse events (all-cause death and heart failure hospitalization) in patients with transcatheter aortic valve replacement. CI, confidence interval; ECV, extracellular volume.

In the five studies, only patients who underwent TAVR were included (*n* = 807). We performed the OR meta-analysis to investigate the prognostic value of CT-ECV in these patients. A meta-analysis demonstrated that a higher CT-ECV was significantly associated with a higher composite adverse events rate (OR = 4.85 per unit increase; 95% CI: 3.26–7.21; *P* < 0.001; *I*^2^ = 0%; *P* for heterogeneity = 0.49) (*Figure 4*).

### Prognostic value of computed tomography extracellular volume in relation to imaging technique, institutional location, and follow-up duration

We performed separate OR meta-analyses to evaluate the prognostic value of CT-ECV across different factors. First, we performed the OR meta-analysis that included single-energy CT^20,21^ and dual-source CT^16–19^ separately. A meta-analysis showed that a higher CT-ECV was significantly associated with a higher composite adverse events rate in single-energy CT (OR = 5.71 per unit increase; 95% CI: 1.99–16.38; *P* = 0.001; *I*^2^ = 25%; *P* for heterogeneity = 0.25) and in dual-source CT (OR = 4.34 per unit increase; 95% CI: 2.89–6.51; *P* < 0.001; *I*^2^ = 0%; *P* for heterogeneity = 0.44) (see Supplementary material online, *Figure S2*).

Next, we analysed studies conducted in Japan^18,20,21^ separately from those conducted in non-Japanese^16,17,19^ institutions. A meta-analysis showed that a higher CT-ECV was significantly associated with a higher composite adverse events rate in Japanese institutions (OR = 4.25 per unit increase; 95% CI: 2.02–8.94; *P* < 0.001; *I*^2^ = 13%; *P* for heterogeneity = 0.32) and non-Japanese institutions (OR = 4.67 per unit increase; 95% CI: 3.01–7.26; *P* < 0.001; *I*^2^ = 0%; *P* for heterogeneity = 0.37) (see Supplementary material online, *Figure S3*).

Finally, we assessed the difference in prognostic value between studies with longer^18,19^ and shorter follow-up periods.^16,17,20,21^ A meta-analysis showed that a higher CT-ECV was significantly associated with a higher composite adverse events rate in studies with longer follow-up periods (OR = 3.36 per unit increase; 95% CI: 2.01–5.62; *P* < 0.001; *I*^2^ = 25%; *P* for heterogeneity = 0.71) and in studies with shorter follow-up periods. (OR = 6.28 per unit increase; 95% CI: 3.67–10.75; *P* < 0.001; *I*^2^ = 0%; *P* for heterogeneity = 0.69) (see Supplementary material online, *Figure S4*).

## Discussion

Our investigation, involving 902 participants from six studies, represents the first comprehensive systematic review and meta-analysis of CT-ECV to assess its prognostic value in patients with severe AS scheduled for AVR. Computed tomography extracellular volume may serve as a valuable tool for preoperative CT planning by evaluating the risk of adverse events in these patients. Our analysis also demonstrated that CT-ECV was significantly associated with composite adverse events, even when limited to patients planned for TAVR. Additionally, the OR meta-analysis indicated that CT-ECV was significantly associated with composite outcomes in patients who underwent either single-energy or dual-source CT.

### Clinical significance of computed tomography extracellular volume assessment

Computed tomography extracellular volume offers a non-invasive and reliable method for assessing myocardial fibrosis and provides crucial prognostic information for patients undergoing AVR. While myocardial biopsy remains the definitive method for identifying myocardial fibrosis, its invasive nature presents challenges. Consequently, non-invasive imaging is expected to become increasingly crucial for evaluating fibrosis. Recent improvements in non-invasive imaging technologies, including CMR and CT, allow for quantitative myocardial fibrosis analysis.^22^ While CMR is typically employed for measuring ECV, using CT angiography to assess ECV offers multiple benefits for patients undergoing TAVR. Recent meta-analyses showed solid correlations between ECV measurements from CT angiography and those from CMR,^22^ and the quantification of CT-ECV has been validated by comparing it with histological evaluations of myocardial fibrosis.^23^ While CMR allows for its detection, logistically, it is rarely performed in the routine clinical care of patients with severe AS. In addition, CMR is not suitable for patients with paramagnetic metal implants or specific cardiac devices. In contrast, CT angiography is faster and generally more manageable for critically ill patients. Importantly, CT angiography is already a standard part of the pre-TAVR evaluation, aiding in the sizing of the aortic valve and the vascular assessments required around the procedure.^24^ Additionally, the measurement of mean CT-ECV was reported to have high intraobserver consistency,^17,23–25^ and adding CT-ECV protocol requires only an additional delayed CT scan, with minimal additional radiation exposure. Our current study showed that only 3–6 min of additional procedure time were required.^16–21^ Previously, renal dysfunction, atrial fibrillation, high Society of Thoracic Surgeons score, respiratory dysfunction, severe ventricular dysfunction, cardiac damage assessed by troponin, coronary artery disease, frailty, and age were known as the prognostic factors after TAVR.^9,26,27^ Computed tomography extracellular volume derived from routine CT scans adds useful prognostic information on these factors, appropriately stratifying the patients after TAVR. Therefore, incorporating CT-ECV could quickly become standard practice in pre-AVR evaluations.

### The mechanism of computed tomography extracellular volume in predicting outcomes for aortic valve replacement candidates

Computed tomography extracellular volume offers different meanings from the conventional severity of AS, as determined in transthoracic echocardiogram and CT. Five of six studies^16–18,20,21^ compared the aortic valve area and mean pressure gradient (PG) between high and low ECV groups. No differences in the aortic valve area were observed in any of the studies. In the study by Tamarappoo *et al*.,^17^ which focused on low-flow and low-gradient AS, the mean PG was significantly lower in patients with high ECV values (ECV > 33%: 25.2 ± 8.3 mmHg vs. ECV ≤ 33%: 28.8 ± 7.0 mmHg, *P* < 0.01). This could be partly explained by the lower LV ejection fraction (LVEF) in the high ECV group (44.9 ± 18.0% vs. 54.0 ± 17.6%, *P* < 0.01), indicating that a reduction in mean PG could be due to impaired systolic function in the high ECV group. The other four studies found no differences between CT-ECV and mean PG.^16,18,20,21^ Peak PG was compared between high and low ECV groups in three studies,^17,18,20^ but no differences were observed. These results suggest that CT-ECV could be a significant prognostic predictor regardless of conventional parameters of AS severity, including aortic valve area, mean PG, and peak PG.

Higher CT-ECV is associated with poor prognosis in patients undergoing AVR for severe AS by reflecting greater myocardial fibrosis and myocardial damage.^16,28^ Increased myocardial fibrosis is associated with markers of myocardial injury and decompensation, such as elevated troponin T and pro-brain natriuretic peptide levels.^16,29,30^ The degree of myocardial damage, as indicated by higher ECV values, may be proportional to the degree of adverse LV remodelling and dysfunction.^30^ In fact, our meta-analysis demonstrated that CT-ECV is associated with LV systolic function, which is the prognostic factor of AVR candidates. In four of the five studies comparing patient backgrounds between the high ECV and low ECV groups,^16–18,20,21^ except for the study by Ishiyama *et al*., LVEF was lower in the high ECV group. Although there was no significant LVEF difference in the study by Ishiyama *et al*.,^20^ LVEF was numerically lower in the high ECV group. Additionally, Koike *et al*.^19^ showed a significant correlation between CT-ECV and LV global longitudinal strain, a more sensitive indicator of LV systolic function than LVEF. Thus, the poor prognosis in the high CT-ECV group is thought to reflect a decrease in LV systolic function due to advanced myocardial fibrosis and damage.

Additionally, cardiac amyloidosis, which coexists with ∼16% of patients with severe AS, may also have a significant prognostic impact.^31^ Aortic stenosis with cardiac amyloidosis has a worse clinical presentation and a trend towards a worse prognosis.^32,33^ Our recent meta-analysis showed that CT-ECV had a high diagnostic accuracy for cardiac amyloidosis, with a sensitivity of 92.8% (95% CI: 86.7%–96.2%), specificity of 84.8% (95% CI: 68.6%–93.4%), and area under the summary receiver-operating characteristic curve of 0.94 (95% CI: 0.88–1.00).^22^ This analysis indicates that elevated CT-ECV could also be caused by cardiac amyloidosis, which is a poor prognostic factor in patients with severe AS. Further investigation is needed to elucidate the mechanism by which high CT-ECV affects prognosis, and it may be necessary to consider early intervention in cases with high ECV values to improve prognosis. Importantly, since CT-ECV measurement is simple and can be routinely performed in cases considered for TAVR, it can potentially be a handy prognostic indicator in the current situation of a dramatic increase in AVR cases.

### Study limitations

First, the papers in the meta-analysis are characterized by relatively small sample sizes and mainly feature retrospective and case-control designs, leading to varied results. A wide range of 28–33% was observed for the cut-off value of ECV in predicting adverse events (see Supplementary material online, *Table S3*). Various factors could cause the heterogeneity of CT-ECV in severe AS. Patient-related factors include comorbidities like hypertension and diabetes that can influence ECV. Methodological differences also play a role; variations in CT scanner types, contrast doses, and imaging timing can affect CT-ECV measurements. Recognizing these factors is crucial for accurately interpreting CT-ECV in clinical practice. Second, the inclusion criteria varied significantly across the studies, leading to a non-negligible risk of selection bias. Third, the predominance of studies from Japan (three out of six) may limit the generalizability of our findings. However, it is notable that the association of CT-ECV with all-cause mortality and HF hospitalization was consistent across studies from both Japan and other countries (see Supplementary material online, *Figure S3*). This consistency suggests that the prognostic value of CT-ECV is applicable across diverse populations. Nevertheless, further research with larger, more diverse cohorts is needed to confirm these findings and enhance generalizability. Fourth, we should consider the influence of the length of the follow-up period on the observed differences in outcomes between the included studies. To investigate this, we performed a subgroup analysis comparing studies with longer follow-up periods with those with shorter follow-up periods. The results were consistent across both groups. Finally, we also need to address the emerging photon-counting CT technology, which enables precise material characterization and holds the potential for identifying specific causes of myocardial interstitial expansion.^34,35^ Although this technology is promising, its utility in patients with severe AS and its routine adoption in clinical practice remain uncertain. In contrast, the conventional evaluation of CT-ECV can be seamlessly incorporated into routine pre-TAVR CT scans, ensuring its clinical relevance and accessibility.

## Conclusions

This study is the first comprehensive systematic review and meta-analysis of CT-ECV to assess its prognostic value in patients with severe AS scheduled for AVR. Considering its high prognostic, predictive ability and versatility, CT-ECV can be used for postoperative risk stratification in patients with severe AS.

## Supplementary Material

oeaf007_Supplementary_Data

## Data Availability

The data underlying this article will be shared on reasonable request to the corresponding author.

## Lead author biography

Jin Kirigaya is a cardiologist at the Yokohama City University Medical Center in Yokohama, Japan. He serves as a physician and clinical researcher in the Cardiac Intensive Care Unit (CICU), focusing on cardiovascular emergencies, cardiovascular imaging, and catheter interventions.
