# Supplementary material for: Prognostic value of computed tomography-derived myocardial extracellular volume in aortic stenosis: a meta-analysis of all-cause mortality and heart failure hospitalization
Source: Eur Heart J Open. 2025 Jan 25;5(1):oeaf007. doi: 10.1093/ehjopen/oeaf007 (PMC11795651; doi:10.1093/ehjopen/oeaf007)
Supplement: oeaf007_Supplementary_Data [file oeaf007_supplementary_data.docx]

**SUPPLEMENTAL MATERIAL**

**Table S1.** Search strategy for Embase and PubMed databases.

| *PubMed* *(31)* | *#1 (computed tomography [Title/Abstract] OR CT[Title/Abstract])*  *#2 (extracellular volume [Title/Abstract] OR ECV[Title/Abstract] OR CT-ECV[Title/Abstract])*  *#3 (aortic stenosis [Title/Abstract] OR AS[Title/Abstract])*  *#4 (#1 AND #2 AND #3)* |
| --- | --- |
| *Web of Science (79)* | *#1 TS = (computed tomography OR CT)*  *#2 TS = (extracellular volume OR ECV OR CT-ECV)*  *#3 TS = (aortic stenosis OR AS)*  *#4 #1 AND #2 AND #3* |
| *EMBASE (27)* | *TITLE: (computed tomography OR CT) AND*  *(extracellular volume OR ECV OR CT-ECV) AND*  *(aortic stenosis OR AS)* |

**Table S2.** Newcastle-Ottawa quality assessment form for case-control studies

| Study | Selection  (Max=4) | Comparability  (Max=2) | Exposure  (Max=3) | Total Score |
| --- | --- | --- | --- | --- |
| Tamarappoo_2020 | 3 | 2 | 3 | 8 |
| Suzuki_2021 | 3 | 1 | 3 | 7 |
| Ishiyama_2013 | 4 | 2 | 3 | 9 |
| Takahashi_2021 | 3 | 2 | 3 | 8 |
| Vignale_2023 | 4 | 2 | 3 | 9 |
| Koike_2023 | 3 | 2 | 3 | 8 |

**Table S3.** Summary of definitions for events and ECV cut-off value, and confusion matrix

| **Author year** | **Follow-up period (months)** | **Definition of adverse events** | **ECV cut-off value, %** | **The choice of methods in determining the cut-off value of CT-ECV** |
| --- | --- | --- | --- | --- |
| Tamarappoo_2020 | 14 (0–29) | all-cause death and hospitalization due to heart failure | 33 | Liu index |
| Suzuki_2021 | 23 (21‒39) | all-cause death and hospitalization due to heart failure | 28 | Median |
| Ishiyama_2013 | 14 ± 8 | all-cause death and hospitalization due to heart failure | 32 | Median |
| Takahashi_2021 | 12 (6–16) | all-cause death and hospitalization due to heart failure | 33 | Youden index |
| Vignale_2023 | 13 (11–15) | all-cause death and hospitalization due to heart failure | 31 | Youden index |
| Koike_2023 | 16 (12–22) | all-cause death and hospitalization due to heart failure | 29 | Median |

ECV = extracellular volume.


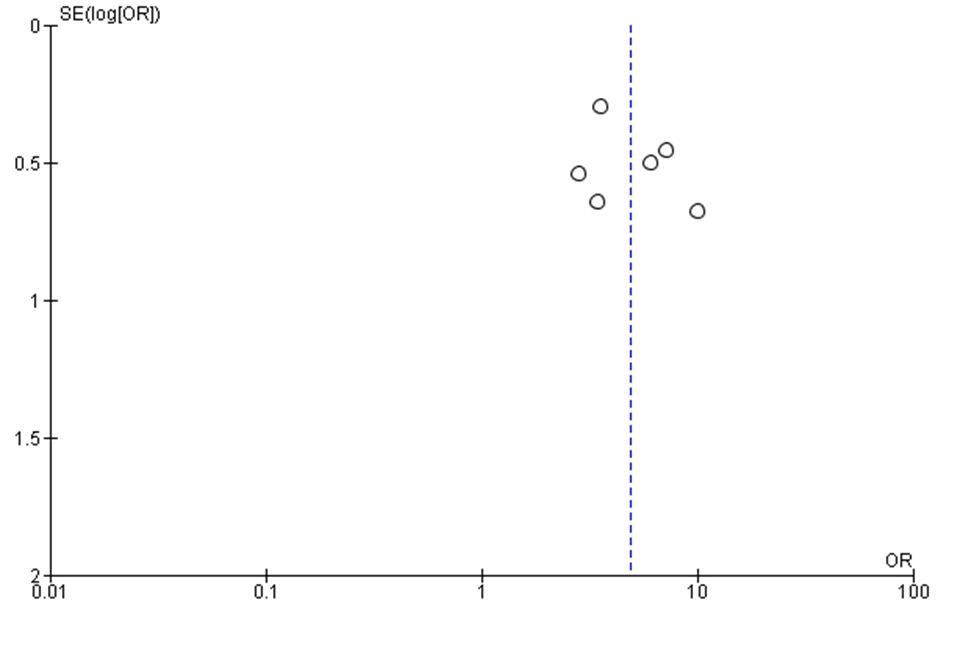


**Figure S1.** Funnel plot of the odds ratio for the composite endpoint in patients with AS (six studies)

Rank Correlation Test for Funnel Plot Asymmetry

Kendall's tau = 0.3333, p = 0.4694

 **Figure S2.** The pooled odds ratio of abnormal CT-ECV for predicting composite adverse events (all-cause death and heart failure hospitalization) in patients who underwent single energy CT and dual-source CT. CI = confidence interval, CT = computer tomography, ECV = extracellular volume.

**Figure S3.** The pooled odds ratio of abnormal CT-ECV for predicting composite adverse events (all-cause death and heart failure hospitalization) in studies conducted at Japanese or non-Japanese institutions.

CI = confidence interval, CT = computer tomography, ECV = extracellular volume.

**Figure S4.** Supplemental Figure 4. The pooled odds ratio of abnormal CT-ECV for predicting composite adverse events (all-cause death and heart failure hospitalization) in studies with longer or shorter follow-up periods.

CI = confidence interval, CT = computer tomography, ECV = extracellular volume.
